# Proposed Prediction Model and Nomogram for Systemic Complications in Patients Undergoing Free Flap Head and Neck Reconstruction

**DOI:** 10.3389/fsurg.2021.771282

**Published:** 2021-12-14

**Authors:** John-Patrik M. Burkhard, Roland Giger, Markus B. Huber, Benoît Schaller, Ayla Little, Sherin Khalil, Dominique Engel, Lukas M. Löffel, Patrick Y. Wuethrich

**Affiliations:** ^1^Department of Anaesthesiology and Pain Medicine, Inselspital, University Hospital Bern, University of Bern, Bern, Switzerland; ^2^Department of Cranio-Maxillofacial Surgery, Inselspital, Bern University Hospital, University of Bern, Bern, Switzerland; ^3^Department of Oto-Rhino-Laryngology, Head and Neck Surgery, Inselspital, Bern University Hospital, University of Bern, Bern, Switzerland

**Keywords:** nomogram, free flap, Clavien-Dindo classification, systemic complications, head and neck surgery

## Abstract

Postoperative complications in head and neck surgery are well-known, but a predictive model to guide clinicians in free flap reconstructions has not been established. This retrospective single-center observational study assessed 131 patients who underwent ablative surgery and received free flap reconstruction. Primary endpoint was the occurrence of systemic complications (PSC). Secondary endpoint was the generation of a nomogram of complications according to the CDC classification. In the ordinal regression model, postoperative administration of furosemide [1.36 (0.63–2.11), *p* < 0.0001], blood loss [0.001 (0.0004–0.0020), *p* = 0.004], postoperative nadir hemoglobin [−0.03 (−0.07–0.01), *p* = 0.108], smoking [0.72 (0.02–1.44), *p* = 0.043], and type of flap reconstruction [1.01 (0.21–1.84), *p* = 0.014] as predictors. A nomogram with acceptable discrimination was proposed (Somer's delta: 0.52). Application of this nomogram in clinical practice could help identify potentially modifiable risk factors and thus reduce the incidence of postoperative complications in patients undergoing microvascular reconstruction of the head and neck.

## Introduction

Free flap reconstruction in major head and neck surgery constitutes a high risk for the development of postoperative complications due to its complexity, with two or more distinct surgical sites and long procedure durations (1). Patients undergoing this type of surgery are often of advanced age, are prone to overconsumption of alcohol and/or tobacco and show multiple comorbidities (2). Perioperative morbidity is closely related to risk factors such as advanced age, higher American Society of Anesthesiologists (ASA) classification, noxious substance consumption, at-risk nutritional status with low preoperative albumin and hemoglobin values, long-lasting and complex surgical procedures, intraoperative blood loss, over-liberal fluid administration, and tumor stage (1–6). Any complication will consequently prolong the hospital stay, result in higher costs and increase the mortality rate. Key factors in predicting minor or major, local and systemic postoperative complications are difficult to identify because of the large number of influencing variables (3). The preoperative identification and possible attenuation of high-risk patients and contributing intraoperative factors are critical to reduce the incidence of postoperative complications and their deleterious sequelae. Almost 25% of all patients undergoing free flap reconstruction are reported to have predominantly postoperative cardiac and pulmonary complications (7, 8). However, most studies investigating this matter have primarily focused on preoperative factors and their relationship with postoperative systemic complications (PSCs). The consideration of intraoperative factors and aspects resulting in a validated, comprehensive clinical tool predicting PSC risk is lacking. To assess the prognosis of diagnostic and treatment responses, nomograms are ideally suited to generate individual numerical probabilities for a clinical event for improved personalized medicine.

The aim of this study was to identify predictive preoperative and intraoperative parameters for PSC and to develop a nomogram. Moreover, perioperative predictors for all (local and systemic) complications were analyzed according to the well-validated Clavien-Dindo classification (CDC) (9) to develop an appropriate nomogram for accurate prediction in patients undergoing free flap reconstruction in head and neck surgery.

## Materials and Methods

This retrospective observational study reports a consecutive case series from a single tertiary center. The study was approved by the Ethics Committee of the Canton of Bern, Switzerland, on January 28, 2020 (KEKBE 2019-01824).

### Study Population, Data Collection, and Outcome Measures

Health-related data were collected from 131 consecutive patients undergoing free flap reconstruction in head and neck ablative surgery, including all malignancies of the head and neck area, osteoradionecrosis, and drug-induced osteonecrosis of the jaw from 2014/01 to 2020/01. Relevant information and patient data were extracted from medical records, including paper records and anesthesia protocols from the clinic internal database.

Preoperative data collection included age, sex, preoperative comorbidities (arterial hypertension, chronic obstructive pulmonary disease (COPD), diabetes mellitus, renal insufficiency, diseases affecting the liver and gastrointestinal tract, and alcohol and/or tobacco consumption) and Charlson Comorbidity Index (CCI).

The intraoperative parameters collected included type of surgical intervention, duration of surgery, flap type (osseous vs. non-osseous), total intraoperative administration of intravenous fluids (crystalloids, colloids, and amount of packed red blood cells), blood loss and total amount of vasopressors (norepinephrine and dobutamine) administered.

The postoperative parameters included lowest hemoglobin value within 5 days after surgery (“nadir hemoglobin”), administration of blood products, and administration of intravenous furosemide as a treatment of fluid overload (“diuretics”).

The first endpoint was the incidence and type of PSC. PSCs were defined as serious complications requiring intervention by medication or interventional means. These were categorized into cardiopulmonary and metabolic events. Cardiopulmonary events included cardiac decompensation with heart insufficiency, pulmonary oedema, or myocardial infarction as well as pulmonary embolism. Relevant metabolic events included refeeding syndrome.

As a secondary endpoint, we reported complications according to the CDC of minor (CDC grade I-II) and major complications (CDC grade IIIa-V); for details, see Appendix (9). We aimed to identify independent risk factors for minor and major postoperative complications.

### Anesthetic Procedure and Patient Monitoring

The detailed anesthetic technique has been described previously (6). In brief, all patients received balanced general anesthesia with state-of-the-art monitoring, including invasive blood pressure measurement (cannulation of the radial artery). Hemodynamic management (goal: systolic blood pressure ≥100 mmHg) was mainly carried out with Ringer's lactate solution (10). If the perfusion index of the pulse oximetry curve was >5 and the urine output was between 0.3 and 0.5 ml/kg/h, euvolemia was assumed, and continuous administration of low-dose norepinephrine (0.02–0.05 μg/kg/min) was initiated after consultation with the lead flap surgeon. Additional dobutamine (2–4 μg/kg/min) and colloids were administered, if necessary.

### Surgical Procedure

Surgery was performed in two teams with the maxillofacial/ear, nose and throat (ENT) team performing the ablative surgery and the plastic surgery and maxillofacial team performing the flap harvest, microanastomoses, and flap inset. A preventive surgical tracheostomy was performed in patients with expected postoperative airway obstruction. The resection and if indicated neck dissection has been undergone. The free flap was raised in parallel. These were either osseous or non-osseous free microvascular grafts to reconstruct the defects mainly in the oral cavity, naso-/ hypopharynx, soft tissue or skin. Mainly anterolateral thigh (ALT), radial forearm, superficial circumflex iliac artery perforator (SCIP) or fibula flap, if bone had to be reconstructed, were used. After flap raising, the flap was finally detached once the ablative surgery was completed and the reconstruction of the defect could be started immediately with the insertion and the microanastomoses to the neck vessels. The entire surgical procedure followed in-house standards (6).

### Postoperative Management

All patients were monitored overnight in the post-anesthesia care unit before being transferred to the ward. Patients with non-osseous flaps were mobilized immediately, and those with osseous flaps were mobilized after 5 days. For reconstructions within the oral cavity, nutrition was provided exclusively by a nasal or percutaneous gastric tube until wound healing was assured. A goal of a systolic blood pressure above 100 mmHg was attempted for sufficient flap perfusion (10). Blood pressure drops were treated with 250–500 mL of crystalloids. Furosemide was administered intravenously when there were signs of fluid overload (dyspnea, oedema, or weight gain).

### Statistical Analysis

Based on the Shapiro-Wilk test of normality, continuous data are expressed as the mean and standard deviation for normally distributed variables and as the median and interquartile range otherwise. Categorical data are presented as counts and frequencies. Group comparisons were computed with unpaired *t*-tests and ANOVA for normally distributed continuous variables and with the Kruskal-Wallis rank sum test otherwise. Group comparisons for categorical variables were based on the chi-squared test or Fisher's exact test when the expected frequencies were <5 in some cells.

Risk factors were selected a priori based on their potential association with PSCs or the CDC. For associations with PSCs, we first applied univariable logistic regression of each predictor with the outcome and examined the association of the predictor with the outcome *via* the odds ratio. The univariable logistic regressions were followed by a multivariable binary logistic regression model featuring all potential predictors. In terms of model selection, a parsimonious model was chosen with respect to known preoperative and intraoperative risk factors for surgical revision due to the limited number of events and to avoid overfitting the regression. A backward stepwise selection procedure based on the Akaike information criterion (AIC) was used to identify independent risk factors for PSCs. We examined the ratio of residual deviance to residual degrees of freedom and performed the Hosmer-Lemeshow (11) and Stukel (12) tests to assess the goodness of fit of the final logistic regression model. Prediction skill was quantified by the Brier score and the receiver operating characteristic area under the curve (ROC-AUC).

For associations with the CDC, we fit a proportional odds ordinal regression model and followed the same predictor selection procedure (i.e., backward stepwise selection according to the AIC). The assumption of proportional odds was examined with the Brant test (13), and the goodness-of-fit of the final ordinal regression was assessed with McFadden's *R*^2^, Cox and Snell's *R*^2^, and Nagelkerke's *R*^2^. The individual impact of each predictor on the two outcomes is illustrated with effect plots, where only one predictor at a time is varied while the other predictors are held constant.

Nomograms were used to visualize the final logistic regression model and final ordinal regression model using the rms package (14). We chose the inverse-logit transformation of the linear predictor to depict the probabilities of the outcomes.

A two-sided *p*-value < 0.05 was considered significant in this study. Analyses were performed using the R software environment (R Foundation for Statistical Computing, Vienna, Austria, Version 4.0.2).

## Results

### Reporting of Complications According to Postoperative Systemic Complications

The baseline, intraoperative and postoperative characteristics are presented in Tables 1, 2. Arterial hypertension (*p* = 0.023), COPD (*p* = 0.018), diabetic metabolic syndrome (*p* = 0.048) and postoperative administration of furosemide (*p* = 0.001) were more frequent in patients with PSCs. The total intraoperative administration of vasopressors did not differ between patients with PSCs and those without PSCs (norepinephrine, *p* = 0.058; dobutamine, *p* = 0.272).

**Table 1:** Baseline and clinical variables.

	**Clavien-Dindo grade**				**Relevant systemic complications**		
	**No complications**	**Minor complications** **CDC I-II**	**Major complications** **CDC IIIa-V**	***P***	**No**	**Yes**	***P***
	***N* = 29**	***N* = 56**	***N* = 46**		***N* = 94**	***N* = 37**	
Age, median [IQR], y	64.0 [55.0;72.0]	61.0 [52.8;71.0]	61.0 [55.2;70.5]	0.496	61.0 [54.0;71.0]	64.0 [58.0;72.0]	0.164
Sex				0.025			1.000
Female	7 (24.1%)	27 (48.2%)	12 (26.1%)		33 (35.1%)	13 (35.1%)	
Male	22 (75.9%)	29 (51.8%)	34 (73.9%)		61 (64.9%)	24 (64.9%)	
Alcohol consumption	13 (44.8%)	19 (33.9%)	25 (54.3%)	0.116	40 (42.6%)	17 (45.9%)	0.875
Tobacco consumption	15 (51.7%)	33 (58.9%)	32 (69.6%)	0.277	57 (60.6%)	23 (62.2%)	1.000
Hypertension	7 (24.1%)	25 (44.6%)	17 (37.0%)	0.179	29 (30.9%)	20 (54.1%)	0.023
CHD	1 (3.45%)	4 (7.14%)	6 (13.0%)	0.337	6 (6.38%)	5 (13.5%)	0.291
Cardiac^*^	0 (0.00%)	1 (1.79%)	7 (15.2%)	0.006	0 (0.00%)	8 (21.6%)	<0.001
COPD	4 (13.8%)	10 (17.9%)	10 (21.7%)	0.682	12 (12.8%)	12 (32.4%)	0.018
CKD classification eGFR [mL/min]				0.217			0.475
GFR>89	27 (93.1%)	50 (89.3%)	44 (95.7%)		88 (93.6%)	33 (89.2%)	
GFR 60–89	1 (3.45%)	5 (8.93%)	0 (0.00%)		4 (4.26%)	2 (5.41%)	
GFR 30–59	1 (3.45%)	1 (1.79%)	2 (4.35%)		2 (2.13%)	2 (5.41%)	
Liver/GIT				0.743			0.825
Healthy	26 (89.7%)	51 (91.1%)	42 (91.3%)		85 (90.4%)	34 (91.9%)	
Viral hepatitis	2 (6.90%)	3 (5.36%)	1 (2.17%)		5 (5.32%)	1 (2.70%)	
Alcohol-related hepatitis	0 (0.00%)	1 (1.79%)	0 (0.00%)		1 (1.06%)	0 (0.00%)	
Peptic ulcer	1 (3.45%)	1 (1.79%)	3 (6.52%)		3 (3.19%)	2 (5.41%)	
Diabetes	3 (10.3%)	4 (7.14%)	6 (13.0%)	0.607	6 (6.38%)	7 (18.9%)	0.048

*CHD, coronary heart disease; CKD, chronic kidney disease; COPD, chronic obstructive pulmonary disease; eGFR, estimated glomerular filtration rate; GIT, gastrointestinal tract; IQR, interquartile range; min, minute; mL, milliliters; SD, standard deviation; y, year.*

* *Summarizes arterial hypertension and CHD*.

**Table 2:** Intraoperative and postoperative variables.

	**Clavien-Dindo grade**				**Relevant systemic complications**		
	**No complications**	**Minor complications** **CDC I-II**	**Major complications** **CDC IIIa-V**	***P***	**No**	**Yes**	***P***
	***N*** **=** **29**	***N*** **=** **56**	***N*** **=** **46**		***N*** **=** **94**	***N*** **=** **37**	
Type of reconstruction				0.069			1.000
Non-osseous	25 (86.2%)	43 (76.8%)	29 (63.0%)		70 (74.5%)	27 (73.0%)	
Osseous	4 (13.8%)	13 (23.2%)	17 (37.0%)		24 (25.5%)	10 (27.0%)	
Duration of surgery [min]	511 [450;564]	570 [487;661]	587 [536;677]	0.002	560 [487;636]	571 [495;650]	0.745
Intraop. iv. fluid [total in mL]	1,400 [1,050;2,050]	1,600 [1,250;2,300]	2,175 [1,542;2,552]	0.018	1,750 [1,278;2,388]	1,800 [1,300;2,500]	0.667
Blood loss [total in mL]	500 [300;600]	600 [450;912]	775 [500;1,150]	0.003	600 [400;900]	700 [400;1,150]	0.359
Postop. nadir Hb [g/L]	99.0 (9.75)	93.2 (9.51)	91.9 (9.01)	0.005	94.7 (9.56)	92.4 (10.0)	0.245
Norepinephrine [total in μg]	192 [0.00;857]	232 [0.00;651]	236 [0.00;922]	0.944	180 [0.00;704]	428 [104;997]	0.058
Dobutamine [total in mg]	11.5 [0.00;37.0]	18.0 [0.00;43.0]	26.0 [0.00;44.5]	0.812	24.0 [0.00;45.8]	0.00 [0.00;32.9]	0.272
Diuretics	5 (17.2%)	18 (32.1%)	28 (60.9%)	<0.001	28 (29.8%)	23 (62.2%)	0.001
Charlson Comorbidity Index	8.00 [4.00;9.00]	7.00 [4.00;9.00]	7.00 [5.00;9.00]	0.703	7.00 [5.00;9.00]	7.00 [4.00;9.00]	0.705
Postop. blood administration [total in mL]	0.00 [0.00;0.00]	275 [275;775]	275 [0.00;719]	<0.001	275 [0.00;550]	275 [0.00;550]	0.957

*g/L, grams per liter; Hb, hemoglobin; iv, intravenous; mcg, micrograms; mg, milligrams; min, minutes; mL, milliliters*.

PSCs occurred in 37/131 patients (28%). Of these 37 patients, 3 (8.1%) had pulmonary embolism, 6 (16.2%) had pneumonia, 16 (43.2%) had electrolyte and metabolic derangement requiring therapy (4/16 had refeeding syndrome), 9 (24.3%) had cardiac decompensation resulting in acute heart insufficiency, peripheral oedema, and/or respiratory distress, and 1 (2.7%) had myocardial infarction. Three of these 37 patients (8.1%) suffered from sepsis due to local wound infection and/or central venous catheter infection. Seven patients (18.9%) suffered from more than one PSC.

**Figure 1:**
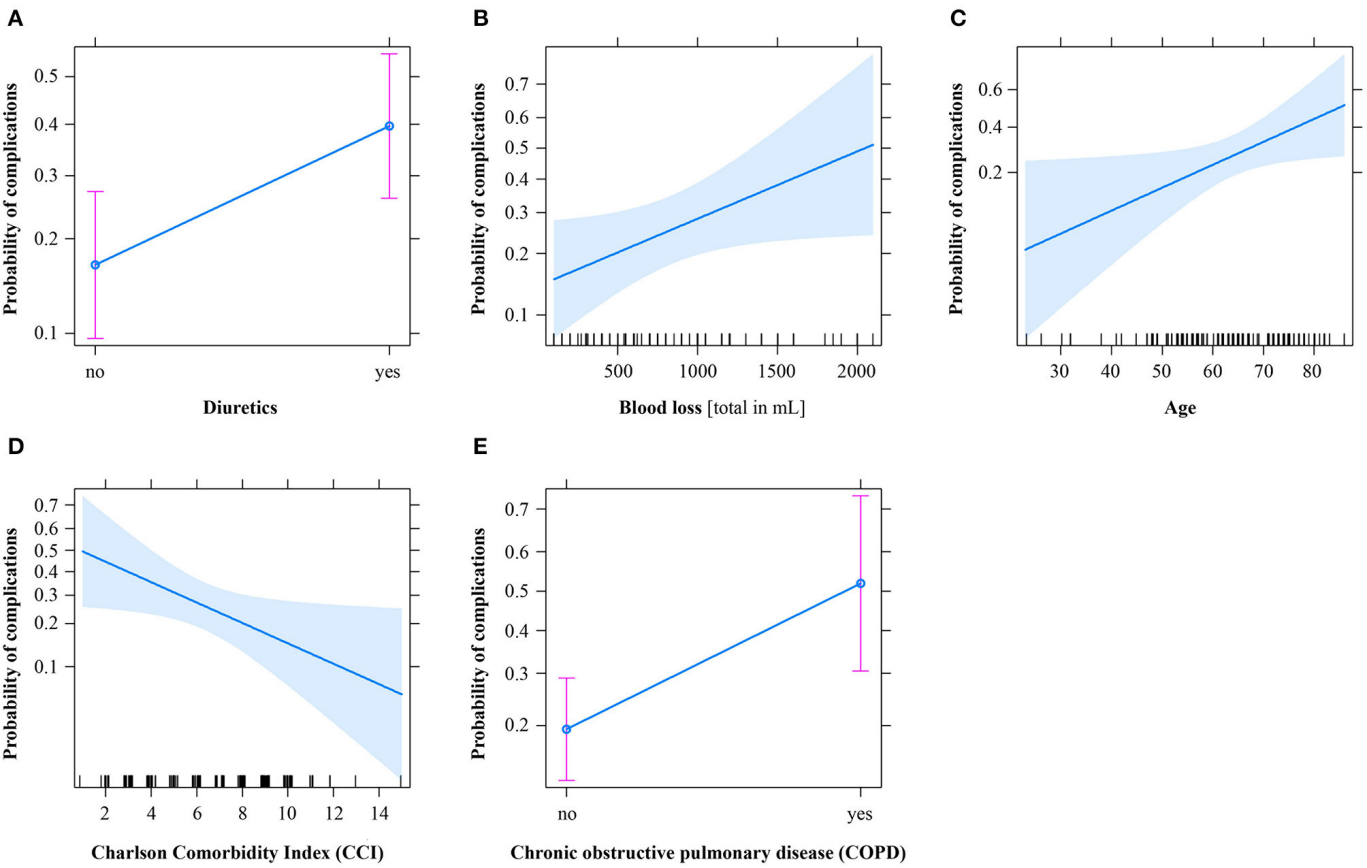
Effect plots of the logistic regression model to predict the outcome “Relevant Systemic Complications.” Solid blue lines denote the mean predictions, and shaded blue bands or pink bars denote the 95% confidence interval. mL, milliliters.

The multiple logistic regression model showed that the variables COPD [0.49 (1.58, 13.5), *p* = 0.006], age [1.05 (1.01, 1.10); *p* = 0.034], CCI [0.83 (0.69, 0.98); *p* = 0.035], intraoperative blood loss [1.0010 (1.00003, 1.0018), *p* = 0.046], and postoperative fluid overload (“diuretics”) [3.29 (1.39, 8.06), *p* = 0.008] were associated with PSCs. The model featured a Brier score of 0.16 and an ROC-AUC of 0.76 (0.69, 0.86) with an acceptable goodness of fit (Hosmer and Lemeshow *P* = 0.14) (Table 3A). The effect plots are presented in Figure 1. We further investigated the protective effect of CCI as suggested by the multiple regression model. The CCI score was highly correlated with age (Pearson's product-moment correlation *r* = 0.51, *p* < 0.0001). Both age and CCI appeared in the final regression model, and the impact of having some patients in this cohort with high CCI scores but no complications (and vice versa) was amplified by having both age and CCI as predictors in the final model. That is, the effect of the CCI on outcome was adjusted for age and the other predictors. Note that the outcome variable and CCI were not associated in the univariable analysis (*p* = 0.705).

**Table 3A:** Logistic regression model summary for the outcome “Relevant Systemic Complications.”

	**OR**	**95% CI**	***p*-value**
Fluid overload/Diuretics	3.29	1.39, 8.06	0.008
Blood loss (total in mL)	1.0009	1.00003, 1.002	0.046
Age at Diagnosis (y)	1.05	1.01, 1.10	0.034
CCI	0.83	0.69, 0.98	0.035
COPD	4.49	1.58, 13.5	0.006

***Model performance metrics:** AUC: 0.76 (95% CI: 0.67-0.86), Brier Score: 0.16, Sensitivity 57%, Specificity 86%, PPV 62%, NPV 84%.*

*CI, confidence interval; CCI, Charlson Comorbidity Index; COPD, chronic obstructive pulmonary disease; mL, milliliters; OR, odds ratio; y, year*.

**Figure 2:**
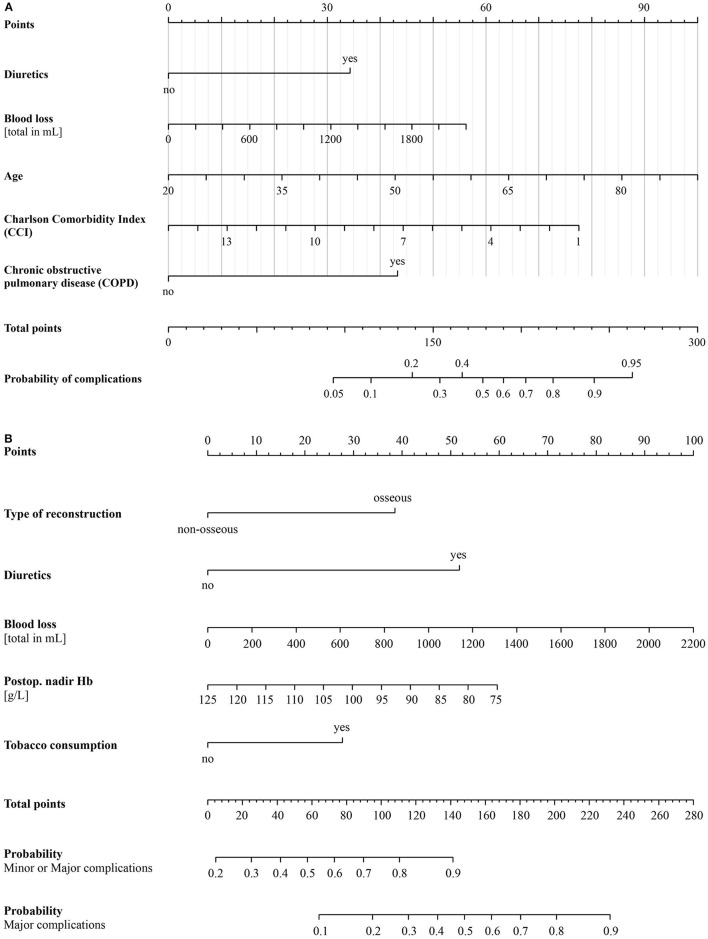
**(A)** Nomogram of the logistic regression model for predicting the outcome “Relevant Systemic Complications.” **(B)** Nomogram of the ordinal regression model for predicting the risk categories of the outcome “Clavien-Dindo classification grade.” g/L, grams per liter; Hb, hemoglobin; mL, milliliters.

Based on the optimized multiple logistic regression model, a nomogram for predicting PSCs was developed and is presented in Figure 2A.

### Reporting of Complications According to the Clavien-Dindo Classification

Minor CDC complications occurred in 56/131 (42.7%) patients, and major CDC complications occurred in 46/131 (35.1%) patients, with 43/46 (93.4%) patients requiring revision surgery in the operating room. Three patients died during their hospitalization (2.3%) due to cardiovascular arrest (2/3) and pulmonary embolism (1/3). Ninety-day mortality was 5.3%, with two additional patients dying from septic shock and another two dying from pneumonia.

Sex (male) (*p* = 0.025), duration of surgery (*p* = 0.002), intraoperative blood loss (*p* = 0.003), total amount of fluid administered (*p* = 0.018), postoperative administration of furosemide (*p* < 0.001), and blood transfusion (*p* < 0.001) were significantly associated with minor and major CDC complications. Vasopressor administration was not associated with CDC complications (norepinephrine, *p* = 0.944; dobutamine, *p* = 0.812) (Tables 1, 2).

**Figure 3:**
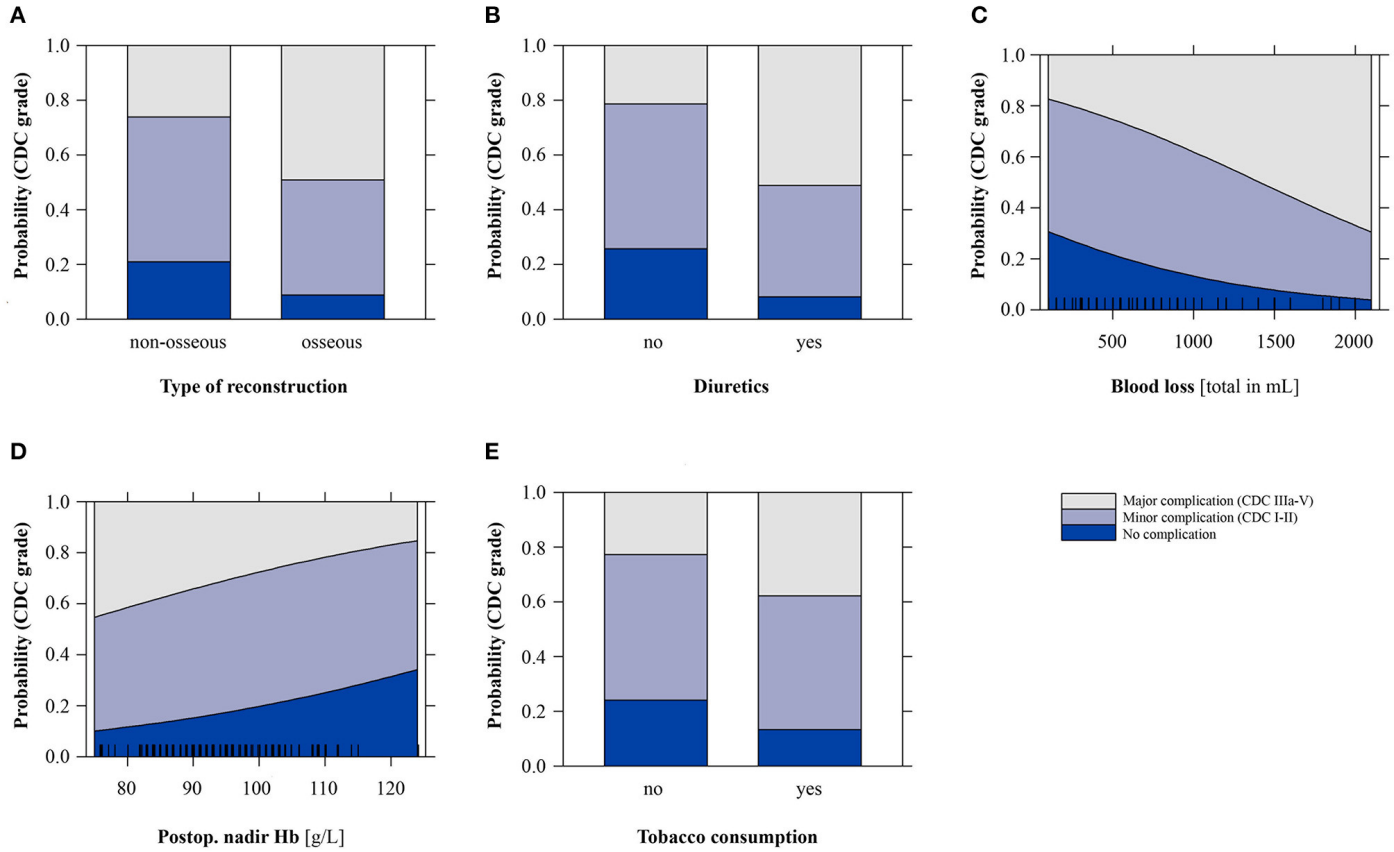
Effect plots of the ordinal regression model for predicting the outcome “Clavien-Dindo classification grade” (CDC; minor CDC: I-II; major CDC: IIIa-V). g/L, grams per liter; Hb, hemoglobin; mL, milliliters.

The following variables remained in the final optimized ordinal regression model (Table 3B): tobacco consumption [0.72 (0.02, 1.44) *p* = 0.043], osseous flap [1.01 (0.21, 1.84), *p* = 0.014], postoperative fluid overload (“diuretics”) [1.36 (0.63, 2.11), *p* < 0.001], and intraoperative blood loss [0.001 (0.0004, 0.0020), *p* = 0.004]. In addition, the postoperative nadir Hb value (g/L) was included, but it was not significant [−0.03 (−0.07, 0.01), *p* = 0.108]. The final model features a Somer's delta measure of ordinal association of 0.52, and the effect plot to predict CDC is presented in Figure 3.

**Table 3B:** Ordinal regression model summary with the outcome “Clavien-Dindo classification grade.”

	**Value**	**95% CI**	***p*-value**
**Intercepts**			
No Complications | Minor Complications	−2.39	−1.41, 6.25	0.227
Minor Complications | Major Complications	−0.02	−3.77, 3.82	0.991
**Coefficients**			
Type of free flap reconstruction [osseous]	1.01	0.21, 1.84	0.014
Fluid overload/Diuretics	1.36	0.63, 2.11	<0.001
Blood loss [total in mL]	0.001	0.0004, 0.0020	0.004
Postop. nadir Hb [g/L]	−0.03	−0.07, 0.01	0.108
Tobacco consumption	0.72	0.02,1.44	0.043

***Model performance metrics:** Pseudo R^2^: 0.14 (McFadden), 0.26 (Cox-Snell), 0.30 (Nagelkerke), Somer's D: 0.52.*

*CI, confidence interval; g/L, grams per liter; Hb, hemoglobin; mL, milliliters; OR, odds ratio; y, year*.

Based on the independent variables identified by the final optimized ordinal regression model, a nomogram was developed to predict the onset of minor and major CDC complications (Figure 2B).

## Discussion

This study highlights the high incidence of patients with PSCs undergoing microvascular free tissue transfer in head and neck reconstruction. Even when applying a validated classification of complications (CDC), the incidence of major complications was 35%. Our results provide evidence that intraoperative blood loss and postoperative administration of diuretics as a surrogate of fluid overload appear to increase PSCs as well as minor and major CDC complications. Furthermore, COPD and/or smoking habits were associated with an elevated risk of PSCs in head and neck microvascular reconstruction. We also developed nomograms for predicting PSCs and minor and major CDC complications. The type of free-flap reconstruction was a significant predictor of CDC complications, illustrating the more holistic approach of this classification.

To date, the occurrence of PSCs associated with perioperative fluid overload in head and neck reconstruction has only been sparsely investigated. In a very recent study, no independent correlation was found between the amount of intraoperative or perioperative fluid administration and the occurrence of PSCs (15). Nevertheless, it is known from other studies that the increased application of crystalloids is associated with flap-specific complications by triggering inflammatory factors, increasing clotting rates, causing excessive oedema in the flap or swelling at the recipient site and mechanically stressing the pedicles (6, 16, 17). It is hypothesized that patients with an underlying cardiac or pulmonary disease would respond negatively to fluid overload. Haughey et al. demonstrated that intraoperative crystalloid administration of more than 7 l was an independent predictor of major systemic complications in patients undergoing head and neck surgery (17). Clark et al. showed similar data based on 185 patients with free-flap reconstruction in head and neck surgery, where intraoperative crystalloid substitution of more than 130 ml/kg per day was an independent predictor of serious medical complications (3). Our study confirmed these findings. The postoperative administration of furosemide as a surrogate of perioperative fluid overload significantly increases the incidence of PSCs, an observation that was confirmed when the CDC system was applied.

The administration of vasopressors remains controversial. However, for the occurrence of PSCs, we could not find a correlation with the administration of norepinephrine or dobutamine in this series, and there is increasing evidence that even in the case of flap-specific complications, the use of vasopressors does not appear to have an influence on outcome (6). To date, no study has identified vasopressors as a cause of increased incidence of systemic complications.

In the case of increased intraoperative blood loss leading to a clinically relevant decrease in hemoglobin concentration, blood products must be administered. Several studies have reported that the transfusion of red blood cells appears to be associated with an increased risk of more severe complications and partial flap necrosis (4, 18) and contributes to significantly more unplanned readmissions (19). Bernard et al. described that patients who received a single unit of packed red blood cells had higher rates of surgical site infections, urinary tract infections, pneumonia, and sepsis/shock, as well as increased composite morbidity and 30-day mortality (20). However, it remains unclear whether this is related to fluid overload, low hemoglobin levels or both. In our study, a low postoperative hemoglobin level was part of the nomogram for predicting CDC complications. In a multicenter study, postoperative anemia was strongly associated with postoperative ischemic events and 90-day mortality (21).

Patients undergoing ablative surgery in the head and neck region are usually of advanced age, are smokers, and have comorbidities. Our PSC nomogram weights postoperative administration of diuretics with >30 points, a CCI of 7 with >40 points, and COPD with 43 points. Furthermore, patients older than 60 years are weighted with ~60 points and relevant blood losses (usually more than 500 ml) with 16 points. This illustrates the fragility of these patients and their need for preoperative optimization.

We were able to show that a total score of ~150 bears a nearly 50% risk of a major CDC complication. A score can easily be reached in the case of a smoker undergoing osseous free flap type reconstruction, with postoperative administration of diuretics and relevant blood loss of more than 1 l. These types of nomograms are useful clinical tools to put a “red flag” on specific cases at high risk for complications.

This study further highlights the importance of adequate patient selection and preparation. Since selection is rarely an option because patients presenting for surgery are what they are, the preoperative optimization of identified high-risk patients may offer the best prospect to reduce the burden of complications. The nomograms generated in this study showed fair discriminative power for use in clinical practice with acceptable safety margins.

The limitations of this study are inherent to its retrospective design and the lack of internal and external validation. Additionally, it is interesting that the CCI shows a counterintuitive value and seems to signify a protective effect. This could be explained by the inhomogeneity of the cohort in terms of a few low CCI patients with complications and a few high CCI patients without complications. Moreover, the CCI score correlated with age (*p* < 0.0001). As both predictors appeared in the final regression model, the impact of having some patients in this cohort with high CCI scores but no complications (and vice versa) was amplified when the effect of CCI on outcome was adjusted for age and the other predictors. Note that the outcome variable and CCI were not associated in the univariable analysis. However, it illustrates the real-life condition of a reference center for this type of surgery.

In conclusion, postoperative fluid overload, increased intraoperative blood loss, smoking, and bony free flap reconstruction were associated with a higher risk of CDC complications. The use of a predictive nomogram in clinical practice could reduce the incidence of postoperative complications and morbidity in patients undergoing head and neck microvascular reconstruction. We also recommend the consistent use of a validated, standardized classification of complications such as the CDC.

## Data Availability Statement

The datasets presented in this article are not readily available as the Cantonal Ethics Committee (Bern, Switzerland) concluded that the data is limited to the registered individuals. Requests to access the datasets should be directed to https://www.gef.be.ch/gef/de/index/direktion/organisation/kek.html.

## Author Contributions

J-PB: conceptualization, data acquisition, methodology, and writing. RG, BS, and LL: reviewing and editing. MH: data curation and analysis, methodology, and statistics. AL: data acquisition. SK: writing. DE: writing and reviewing. PW: conceptualization, reviewing, editing, methodology, and writing. All authors contributed to the article and approved the submitted version.

## Conflict of Interest

The authors declare that the research was conducted in the absence of any commercial or financial relationships that could be construed as a potential conflict of interest.

## Publisher's Note

All claims expressed in this article are solely those of the authors and do not necessarily represent those of their affiliated organizations, or those of the publisher, the editors and the reviewers. Any product that may be evaluated in this article, or claim that may be made by its manufacturer, is not guaranteed or endorsed by the publisher.

## Figures and Tables

**Table A1:** Clavien-Dindo classification of surgical complications (9).

**Grade**	**Definition**
I	Any deviation from the normal postoperative course without the need for pharmacological treatment or surgical, endoscopic, or radiological interventions. Permitted therapeutic regimens are drugs as antiemetics, antipyretics, analgesics, diuretics, electrolytes, and physiotherapy. The grade also includes wound infections opened at the bedside.
II	Requiring pharmacological treatment with drugs other than those permitted for grade I complications. Blood transfusions and total parental nutrition are also included.
III a/b	Requiring surgical, endoscopic, or radiological intervention without (a) / with (b) general anesthesia.
IV	Life-threatening complication (including complications of the central nervous system) that requires management in a high dependency, or intensive therapy unit. - Single organ dysfunction (including dialysis) - Multiorgan dysfunction
V	Death
